# Health-related quality of life in patients with Gilles de la Tourette syndrome at the transition between adolescence and adulthood

**DOI:** 10.1007/s10072-016-2682-y

**Published:** 2016-07-25

**Authors:** Paola R. Silvestri, Flavia Chiarotti, Valentina Baglioni, Valeria Neri, Francesco Cardona, Andrea E. Cavanna

**Affiliations:** 1 Department of Pediatrics and Child Neuropsychiatry, Sapienza University, Rome, Italy; 2 Department of Cell Biology and Neuroscience, National Institute of Health, Rome, Italy; 3 Department of Neuropsychiatry, The Barberry National Centre for Mental Health, BSMHFT and University of Birmingham, 25 Vincent Drive, Birmingham, B15 2FG UK; 4 Sobell Department of Motor Neuroscience and Movement Disorders, Institute of Neurology and University College London, London, UK; 5 School of Life and Health Sciences, Aston Brain Centre, Aston University, Birmingham, UK

**Keywords:** Anxiety, Gilles de la Tourette syndrome, Health-related quality of life, Tics

## Abstract

Gilles de la Tourette syndrome (GTS) is a neurodevelopmental condition characterised by tics and co-morbid behavioural problems, affecting predominantly male patients. Tic severity typically fluctuates over time, with a consistent pattern showing improvement after adolescence in a considerable proportion of patients. Both tics and behavioural co-morbidities have been shown to have the potential to affect patients’ health-related quality of life (HR-QoL) in children and adults with persisting symptoms. In this study, we present the results of the first investigation of HR-QoL in patients with Gilles de la Tourette syndrome at the transition between adolescence and adulthood using a disease-specific HR-QoL measure, the Gilles de la Tourette Syndrome-Quality of Life-Children and Adolescents scale. Our results showed that patients with GTS and more severe co-morbid anxiety symptoms reported lower HR-QoL across all domains, highlighting the impact of anxiety on patient’s well-being at a critical stage of development. Routine screening for anxiety symptoms is recommended in all patients with GTS seen at transition clinics from paediatric to adult care, to implement effective behavioural and pharmacological interventions as appropriate.

## Introduction

Gilles de la Tourette syndrome (GTS) is a neurodevelopmental disorder characterised by multiple motor and phonic tics [1]. The average age at onset of GTS is around 7 years, and a significant reduction of tic severity after adolescence occurs in about one-third of cases [1]. Co-morbid behavioural problems are reported by the majority of patients with GTS, especially attention-deficit and hyperactivity disorder (ADHD), obsessive–compulsive disorder (OCD), anxiety and affective disorders [1]. Clinical studies in children and adults with GTS have shown that both tics and behavioural symptoms can affect patients’ health-related quality of life (HR-QoL) [2]. To date, few data are available about the impact of GTS on HR-QoL at the transition between adolescence and adulthood, a crucial age for patients with tic disorders. We, therefore, set out to investigate HR-QoL and its determinants in a clinical sample of adolescents and young adults with GTS using a disease-specific HR-QoL measure, alongside a comprehensive battery of psychometric instruments [3].

## Methods

### Participants

Twenty-two consecutive patients (5 girls) with a diagnosis of GTS and a mean age of 18 years [age range 15–19 years; standard deviation (SD) 1 year] were recruited from the Child and Adolescent Neuropsychiatry Outpatient Unit of the Sapienza University in Rome, Italy. Each participant/parent provided written informed consent before enrolment. Eight out of the 22 patients had no co-morbid psychiatric disorders, 8 were diagnosed with OCD, 4 with ADHD, and 2 with both OCD and ADHD. The mean age at tic onset was 8 years (SD 3 years). Eighteen patients were on pharmacotherapy (8 with atypical antipsychotics, 1 with typical antipsychotics, 2 with antidepressants, 7 with other medications).

### Procedure

Specialist clinicians with extensive experience in tic disorders collected demographic and clinical information and assessed the severity of tics using the Yale Global Tic Severity Scale (YGTSS) [4], a clinician-rated instrument that assesses the number, frequency, intensity, complexity and interference of motor and phonic tics. YGTSS total tic severity scores range from 0 (no tics) to 50 (extremely severe tics). Each participant was subsequently asked to complete the following psychometric instruments to assess HR-QoL, premonitory urges, and co-morbid behavioural symptoms:

- *Gilles de la Tourette Syndrome*-*Quality Of Life*-*Children and Adolescents scale (C&A*-*GTS*-*QOL)* [5]
  The C&A-GTS-QOL is a 27-item, self-report questionnaire that assesses HR-QoL in young patients with tic disorders, encompassing 4 areas of HR-QoL: psychological, physical/activities of daily living, obsessive–compulsive, and cognitive domains. Each item is rated on a 5-point Likert-type scale and higher scores indicate worse HR-QoL. The instrument includes a Visual Analogue Scale used to express the extent of self-satisfaction about life (higher scores indicate higher satisfaction).
- *Premonitory Urge for Tics Scale (PUTS)* [6]
  The PUTS is a 10-item self-report scale that assesses premonitory urges to tic. Items from 1 to 9 receive a score ranging from 1 (never) to 4 (always). Higher scores indicate the presence of more severe urges.
- *State*-*Trait Anxiety Inventory*-*form Y (STAI*-*Y)* [7]
  The STAI is a 40-item, self-report questionnaire assessing state (STAI-Y1) and trait anxiety (STAI-Y2) in subjects older than 12 years. Items are rated on a 4-point Likert-type scale, and higher scores indicate more severe symptoms.
- *Beck Depression Inventory*-*II (BDI*-*II)* [8]
  The BDI-II is a 21-item, self-report questionnaire assessing depression in subjects older than 13 years. Items are rated on a 4-point Likert-type scale, and higher scores indicate more severe symptoms.

### Statistical analysis

Participants were divided into subgroups based on the following grouping variables: (a) gender (male vs female); (b) tic severity (YGTSS score <10 vs ≥10); (c) premonitory urge severity (PUTS score <25 vs ≥25); (d) anxiety state (STAI-Y1 <75 vs ≥75th centile) and trait (STAI-Y2 <75 vs ≥75th centile); (e) affective symptoms (BDI-II <90 vs ≥90th centile). Differences in GTS-QOL-C&A scores among subgroups were assessed using the Mann–Whitney *U* test. Statistical analyses were performed using the STATA Statistical Software, Version 8.1.

## Results

In our sample, C&A-GTS-QOL total and subscale scores were lower in patients who reported more severe state and trait anxiety (Table 1).

**Table 1:** Health-related quality of life ratings (mean values ± SD) in patients with Gilles de la Tourette syndrome grouped by clinical variables

	C&A-GTS-QOL total	C&A-GTS-QOL psychological	C&A-GTS-QOL obsessional	C&A-GTS-QOL cognitive	C&A-GTS-QOL physical/ADL	C&A-GTS-QOL VAS
BDI-II (*p*)	0.0767	0.1111	**0.0452**	0.4590	0.1718	0.0865
Neg (*n* = 16)	26.56 ± 17.64	29.63 ± 17.10	**19.06** **±** **20.10**	31.44 ± 26.16	24.00 ± 20.45	74.06 ± 17.34
Pos (*n* = 6)	43.00 ± 17.47	47.50 ± 20.00	**44.17** **±** **29.40**	35.67 ± 18.82	39.83 ± 24.64	54.50 ± 22.12
PUTS (*p*)	0.0559	0.4104	**0.0305**	0.0804	0.1714	0.4688
Neg (*n* = 14)	24.93 ± 12.19	31.21 ± 14.86	**15.00** **±** **13.01**	25.86 ± 19.73	21.79 ± 15.03	70.14 ± 23.39
Pos (*n* = 8)	41.75 ± 23.91	40.25 ± 25.34	**45.00** **±** **30.12**	44.38 ± 27.55	39.75 ± 28.84	66.25 ± 14.33
STAI-Y1 (*p*)	**0.0064**	**0.0010**	**0.0106**	0.0662	0.1349	**0.0143**
Neg (*n* = 14)	**22.00** **±** **13.34**	**24.79** **±** **14.00**	**15.00** **±** **18.29**	23.14 ± 15.32	22.36 ± 19.41	**77.50** **±** **15.66**
Pos (*n* = 7)	**46.43** **±** **17.79**	**52.71** **±** **15.91**	**44.29** **±** **26.37**	43.57 ± 25.51	38.71 ± 26.26	**53.86** **±** **20.27**
STAI-Y2 (*p*)	**0.0228**	0.0510	**0.0096**	0.1543	0.1001	**0.0104**
Neg (*n* = 14)	**23.14** **±** **13.36**	28.00 ± 15.68	**14.64** **±** **15.50**	24.50 ± 17.00	21.07 ± 16.99	**77.14** **±** **16.49**
Pos (*n* = 7)	**44.14** **±** **20.90**	46.29 ± 22.17	**45.00** **±** **29.15**	40.86 ± 25.48	41.29 ± 27.77	**54.57** **±** **19.78**

Statistically significant values (*p* ≤ 0.05; Mann–Whitney *U* test) shown in bold

*C&A-GTS-QOL* Gilles de la Tourette Syndrome-Quality Of Life-Children and Adolescents scale, *ADL* activities of daily living, *VAS* Visual Analogue Scale, *BDI-II* Beck Depression Inventory-II; *PUTS* Premonitory Urge for Tics Scale, *STAI-Y1* State-Trait Anxiety Inventory-State Anxiety, *STAI-Y2* State-Trait Anxiety Inventory-Trait Anxiety

Analysis of C&A-GTS-QOL VAS scores confirmed these findings, as patients with lower levels of anxiety consistently reported higher overall satisfaction with life. The negative effects of anxiety symptoms on HR-QoL were particularly significant in the psychological domain, whereas the obsessional domain of HR-QoL was also affected by the presence of more severe urges to tic and affective symptoms. Patients with at least one co-morbid psychiatric diagnosis reported higher C&A-GTS-QOL scores compared to patients with GTS only, although these differences did not reach statistical significance (*p* = 0.09). No significant differences in C&A-GTS-QOL scores were observed between male and female patients, or between patients with lower and higher tic severity.

## Discussion

We conducted the first study focusing on HR-QoL and its clinical correlates in patients with GTS at the transition age between adolescence and adulthood. This is a particularly delicate age for patients with tic disorders, as the persistence of symptoms into early adulthood suggests that the tic disorder might persist throughout life as a potentially stigmatising condition. Interestingly, in our clinical sample, neither gender nor tic severity was associated with decreased HR-QoL, and the impact of premonitory urges and affective symptoms was limited to the obsessional domain of HR-QoL. However, we found a significantly lower report of HR-QoL in patients with higher levels of state and trait anxiety. These results are consistent with findings from previous studies using generic HR-QoL instruments in adult patients with GTS, which highlighted a pervasive effect of anxiety on HR-QoL [9] and showed that anxiety symptoms, as well as affective symptoms, moderate the relationship between tic severity and functional impairment, such that stronger relationships are documented in participants with elevated anxiety or depression [10]. The effects of psychiatric co-morbidities, especially ADHD and OCD, on the HR-QoL of patients within this age group deserve further investigation, based on our preliminary findings [11]. Limitations of the present study include the relatively small sample size (especially with regard to the subgroup of patients with GTS only), referral bias, use of self-report measures and retrospective design. Our findings highlight the crucial role that anxiety symptoms play in influencing the psychological well-being of patients with GTS from adolescence to early adulthood. Routine screening for anxiety symptoms is recommended in all patients with GTS seen at transition clinics from paediatric to adult care, to implement effective behavioural and pharmacological interventions as appropriate.
